# The ReCaPTa study - a prospective out of hospital cardiac arrest registry including multiple sources of surveillance for the study of sudden cardiac death in the Mediterranean area

**DOI:** 10.1186/s13049-016-0309-1

**Published:** 2016-10-19

**Authors:** Youcef Azeli, Eneko Barbería, María Jiménez-Herrera, Gil Bonet, Eva Valero-Mora, Alfonso Lopez-Gomariz, Isaac Lucas-Guarque, Alex Guillen-Lopez, Carlos Alonso-Villaverde, Inés Landín, Pilar Torralba, Ali Jammoul, Jordi Bladé-Creixenti, Christer Axelsson, Alfredo Bardají

**Affiliations:** 1 Emergency Medical System of Catalonia (SEM), 112 Reus, Carrer del pagesos 2, 43204 Reus, Spain; 2 Emergency Department Sant Joan University Hospital, Reus, Spain; 3 Pathology Service, Institute of Legal Medicine and Forensic Science, Catalonia, Spain; 4 Rovira Virgili University, Tarragona, Spain; 5 Nursin Department, Rovira Virgili University, Tarragona, Spain; 6 Cardiology Service, Joan XXIII University Hospital, Tarragona, Spain; 7 Pere Virgili Health Research Institute, Tarragona, Spain; 8 Primary Care Center of Cambrils, SAGESSA, Cambrils, Spain; 9 Internal Medicine Department Sant Joan University Hospital, Reus, Spain; 10 LLevant Clinic Unit, Santa Tecla Hospital, Tarragona, Spain; 11 Primary care area of Tarragona, Catalan Institute of health, Tarragona, Spain; 12 University of Borås, Borås/Va Götaland, Borås, Sweden

**Keywords:** Out-of-hospital cardiac arrest, Sudden cardiac death, Multiple sources of surveillance registry, Cardiopulmonary resuscitation

## Abstract

**Background:**

Cardiovascular diseases are one of the leading causes of death in the industrialized world. Sudden cardiac death is very often the first manifestation of the disease and it occurs in the prehospital setting.

The determination of the sudden cardiac death phenotype is challenging. It requires prospective studies in the community including multiple sources of case ascertainment that help to identify the cause and circumstances of death.

The aim of the Clinical and Pathological Registry of Tarragona (ReCaPTa) is to study incidence and etiology of Sudden Cardiac Death in the Tarragona region (Catalonia, Spain).

**Methods:**

ReCaPTa is a population-based registry of OHCA using multiple sources of surveillance. The population base is 511,662. This registry is compiled chronologically in a relational database and it prospectively contains data on all the OHCA attended by the EMS from April 2014 to April 2017. ReCaPTa collects data after each emergency medical assistance using an online application including variables of the onset of symptoms. A quality control is performed and it permits monitoring the percentage of cases included by the emergency crew. Simultaneously, data from the medico-legal autopsies is taken from the Pathology Center of the area. All the examination findings following a specific protocol for the sudden death study are entered into the ReCaPTa database by one trained person. Survivors admitted to hospital are followed up and their clinical variables are collected in each hospital. The primary care researchers analyze the digital clinical records in order to obtain medical background. All the available data will be reviewed after an adjudication process with the aim of identifying all cases of sudden cardiac death.

**Discussion:**

There is a lack of population-based registries including multiple source of surveillance in the Mediterranean area. The ReCaPTa study could provide valuable information to prevent sudden cardiac death and develop new strategies to improve its survival.

## Background

Every year in Europe 275,000 experience out of hospital cardiac arrests (OHCA) treated by the emergency medical services (EMS); of these only 29,000 survive to hospital discharge [1]. Most of these OHCA take place unexpectedly and suddenly, which makes them even more difficult for the health care system to treat.

OHCA records have been used to make global sudden cardiac death (SCD) comparisons between different countries [2]. Estimates for the rate of SCD vary widely depending on the sources of information used, the methods of case ascertainment, the definitions of Sudden death (SD) and interregional differences [3].

Mortality statistics in high-income countries are based on cause of death certificates in more than 99 % of cases [4]. The accuracy of death certification depends on the quality of postmortem investigations and the coding procedures used during the registration process [5]. Death certificates are also known to overestimate SCD rates [6].

The leading cause of SCD is produced in the setting of chronic or acute ischemic heart disease [7]. The study of cardiovascular risk factors has a long and proven history in Catalonia [8]. In addition, primary prevention strategies have a high cost and are limited to a few numbers of patients. In spite of all these efforts and the improvements in care, the rate of SCD does not seem to have changed in recent years as it has been expected and survival rates still remain poor [3].

SCD is generally defined as a sudden and unexpected pulseless event. To obtain an accurate estimate of its rates non cardiac conditions need to be excluded before the occurrence of a primary cardiac cause can be confirmed [9]. Identifying accurately SCD requires prospective studies that draw on with multiple sources of surveillance for case ascertainment. There have been a few [10–12] but none have been conducted for the Mediterranean area.

A comprehensive OHCA registry for studying SCD must include all possible sources of information. In the present study, we provide detailed Utstein data [13] taken from the EMS, which include symptoms prior to the collapse, data from the medico-legal autopsies performed, data from the medical background of the patient and data from the follow up of the patients admitted alive to hospital. Once we had obtained these data, we designed the clinical and pathological registry (ReCaPTa study).

## Methods

### Study design

ReCaPTa is a population-based registry of OHCA using multiple sources of surveillance. This study prospectively contains data on all the OHCA attended by the EMS from April 2014 to April 2017.

All OHCA assessed or treated by the EMS are included. All resuscitation attempts by the EMS, bystander CPR or bystander automated external defibrillator (AED) are also included.

Cardiac arrest (CA) is defined as a cessation of cardiac mechanical activity and is confirmed by the absence of signs of circulation [14]. Place of residence, public places, long term care facilities and primary care centers are regarded as being out of hospital.

### Aim

#### Primary endpoint

To study incidence and etiology of sudden cardiac death in the Tarragona region (Mediterranean area).

#### Secondary endpoints

To study epidemiological, clinical and pathological variables of Sudden Cardiac Death.

To study injuries from chest compressions during resuscitation and to investigate risk factors associated with their genesis.

### Study area

**Fig. 1:**
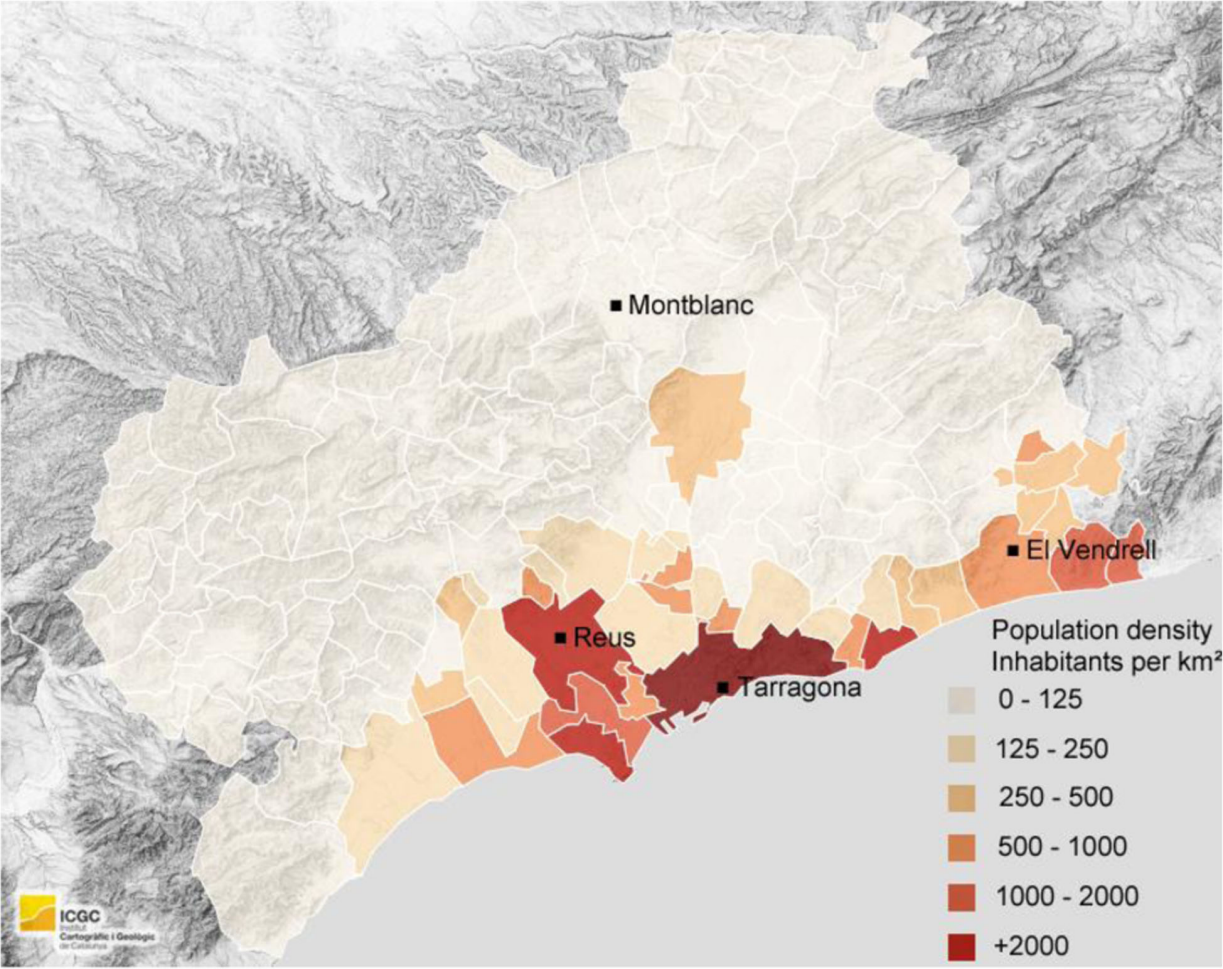
Camp de Tarrag﻿ona population density and ALS units situation

The Camp de Tarragona is a coastal region situated in the western Mediterranean and is made up of six districts. It has an area 2703.3 km^2^ and 511,662 inhabitants. Its population density in 2014 was 190.7 hab/km^2^ according the Statistical Institute of Catalonia [15]. This region has six municipalities with > 20,000 inhabitants, most of them located close to the coast (Fig. 1) [16]. During the summer some municipalities experience a fivefold increase in population. Forty six percent of the population is concentrated in two main municipalities, which together form the second largest metropolitan area in Catalonia. The median age of the population is 40 years and 50.49 % are men.

### Organization of the emergency medical system

The Catalan EMS is a two-tier emergency response system that works as a part of the public health system. Is the only EMS working in Tarragona and serves the entire population.

**Fig. 2:**
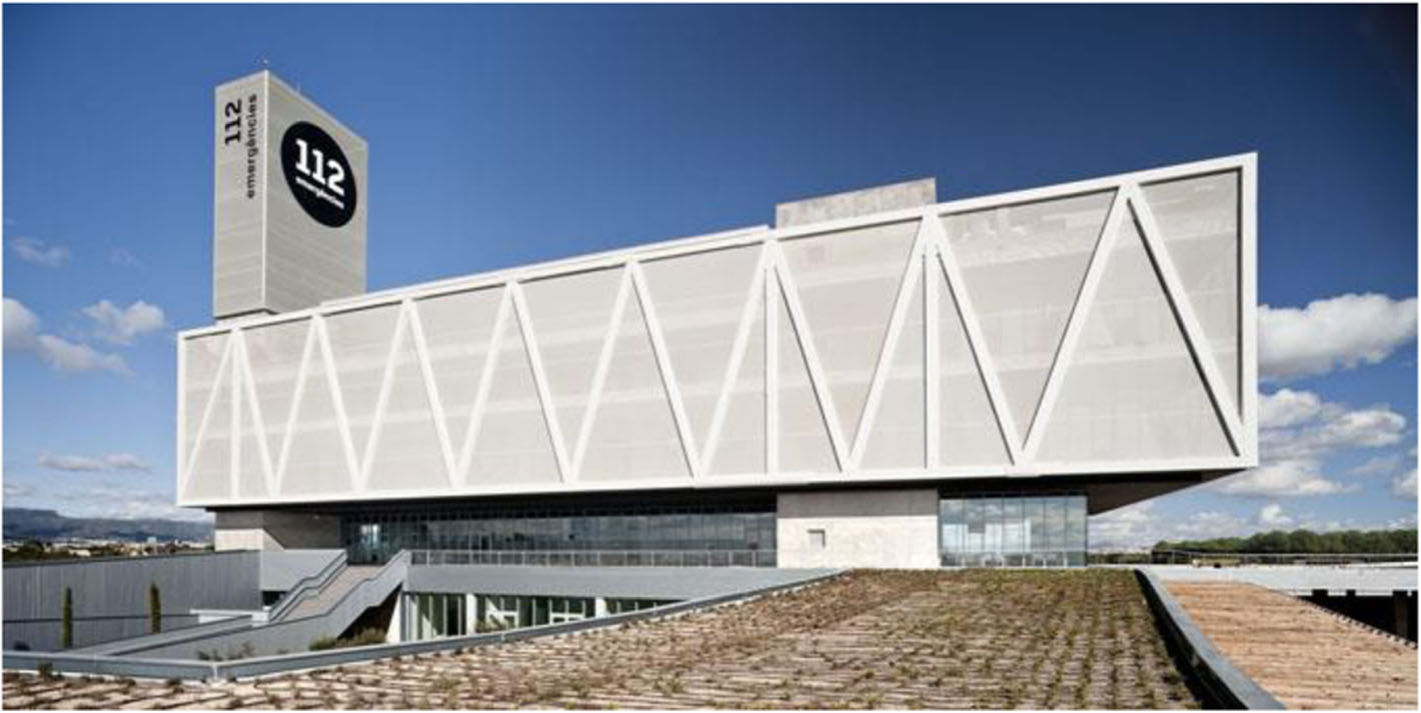
112 dispatch center

All the emergency services in the region are coordinated by a single dispatch center (Fig. 2). Urgent and non-urgent health calls are made using the 112 or 061 telephone numbers. The calls are attended by a team of nurses and physicians who ask a series of simple questions following guidelines aimed at the early recognition of cardiac arrest [17]. When cardiac arrest is suspected, two ambulances are activated, a basic life support (BLS) ambulance and an advanced life support (ALS) ambulance.

When this study commenced, the EMS possessed 46 terrestrial ambulances and an advanced air ambulance. There were 42 BLS rescue units staffed with two ambulance technicians and four ALS rescue units staffed with one physician, one nurse and one ambulance technician.

### Institute of legal medicine and forensic science of Catalonia (ILMFSC)

In Spain in accordance with the law, a forensic autopsy is required for all violent deaths and in which there is an unknowned cause of death. This includes sudden and unexpected natural deaths in non-hospitalized persons [18].

The Pathology Center in the region is based in the city of Tarragona. A complete autopsy is performed, as is a necropsy that also includes the clinical information available relating to the circumstances of death and the toxicological and histopathological findings. All examinations follow the ILMFSC protocol for the study of SD [19] and are coded in accordance with the international code disease ICD-10. Patients presenting a non-traumatic CA are included in the study of lesions due to cardiopulmonary resuscitation (CPR). Injuries during resuscitation are evaluated using a worksheet based in the Englund and Kongstad protocol along with a study of the distribution of the lesions and the anthropometric variables [20]. All the data are entered into the forensic ReCaPTa database by one trained person.

### Hospital net

The Camp de Tarragona region has three hospitals equipped with intensive care units (ICU) able to admit patients who have suffered an OHCA. There is only one hospital in Tarragona city equipped with a catheterization laboratory able to perform cardiac catheterization 24h a day on every day of the year.

When a cardiac arrest due to a coronary cause is suspected, the resuscitated patient is transferred to a hospital with cardiac catheterization capacity. The diagnostic test and the treatment are decided by the physician in charge and in accordance with the current guidelines [21, 22].

### The data from the EMS is collected using an online Web Application

OHCAs are reported continuously by the ALS crew through an online application in the computer of each ambulance base or by portable devices such as a mobile phone. All data is collected in accordance with the Utstein recommendations. The application is linked to the Swedish Registry of Out-Of-Hospital-Cardiac-Arrests. The application was originally created for a randomized trial on registry patients to measure the effectiveness of passive leg raising during OHCA [23] and collects specific data for the ReCaPTa Study relating to the onset of symptoms prior to collapse.

### The quality control of the EMS database

Quality control of the data is performed by the study’s researchers in collaboration with individuals specially trained in handling medical records. The analysis is performed in accordance with the Utstein variables and the literal meaning of text report. All cases registered by the dispatch center codified with one of the following CIE-9 codes: 798.1 (instantaneous death), 798.9 (Unattended death), 427.5 (cardiac arrest) and 427.41 (ventricular fibrillation), are checked. All cases attended by ALS are identified and the digitalized medical report are obtained from the EMS record system and reviewed. All the BLS manual paper records are collected and reviewed as well. OHCA data are also cross-checked to ensure that all the non-reported treated OHCA in the EMS database are included.

The percentage of cases included in the online database monitoring is periodically communicated to the emergency teams in order to increase the percentage of cases reported to the register.

### Registration procedures

The Clinical and Pathological Registry of Tarragona is compiled chronologically in a relational database. All the OHCA attended by the EMS are included. When EMS personnel perform chest compressions or defibrillation it is recorded as a resuscitation attempt by EMS personnel [13]. Bystander CPR or Bystander automated external defibrillator (AED) are also included.

The ILMFSC includes all cases involving autopsies carried out in accordance with the aforementioned criteria. Cases are crossed and matched between the EMS and ILMFSC databases and data from both sources are entered in order of date of death on a single database. Patients who suffered SD and were not initially attended by the EMS are also included.

Survivors admitted to hospital are followed up and their variables are collected at each hospital by researchers involved in the study. The primary care researchers analyze the digital clinical records in order to obtain background medical data.

An adjudication process will be carried out to appoint three researchers on the study to review all the available data including circumstances of death, medical records and autopsy records with the aim of identifying all cases of SCD. The study uses the World Health Organization’s criteria for SCD [24]. SCD is defined as an unexpected sudden, pulseless condition of cardiac etiology, occurring within one hour of symptom onset in witnessed cases and within 24 h if unwitnessed. Survivors of sudden cardiac arrest are also included as SCD cases.

All hospital records, medico-legal autopsy reports and medical backgrounds will be reviewed in order to study the causes of death. Validation of acute coronary death was based on symptoms, enzymes, and series of ECG findings, coronary angiograms and necropsy findings.

Data quality is controlled at all times during the collection data process. When all the data for a given case have been collected, the case is marked as definitively closed. When the information is incomplete or pending, the case is marked as pending review.

### Study protocol variables

#### Variables collected by EMS

. Ambulance journal number, district, ambulance unit, date.

. Early suspension of life support maneuvers on medical orders: yes (medical background, age, lesions incompatible with life, do not resuscitation orders, others), no.

. Time of: CA, call to dispatch center, ambulance alert, ambulance start, arrival at address, arrival at patient’s side, CPR start, first defibrillation, return of spontaneous circulation (ROSC), start of transport to hospital, arrival at hospital.

. Patient data: age, gender.

. Data on the OHCA incident:

- Place of occurrence (home, public place, ambulance, health center…).
- Witnessed: yes (bystander, ambulance staff), no.
- CPR before ambulance arrival: yes (layperson, healthcare workers, other public services), no.
- If CPR before ambulance: defibrillation Y/N (number), ventilation Y/N, chest compression Y/N.
- Status on arrival of ambulance: unconscious Y/N, breathing (normal, agonal, no), pulse Y/N.
- Semi-automatic defibrillator: Y/N.
- Heart rhythm: (VF, VT, PEA, asystole).
- Suspicious reason for CA: Medical: cardiac cause, pulmonary embolism, pulmonary disease, neurological cause/ Non-medical: trauma, suicide, toxics, drowning/other reason/not recorded
- Treatment: (chest compression, mechanical compression, ventilation, intubation, initial end tidal CO2 (number), defibrillation (number), epinephrine, atropine, amiodarone, hypothermia.
- Result: ROSC, to hospital Y/N (if yes, which hospital).
- On arrival at hospital: pulse-giving rhythm Y/N.
- Name of hospital.
- Symptoms before CA: dyspnea, abdominal pain, thoracic pain, infection suggestive symptoms, dizziness, syncope, cough, sphincter relaxation, palpitations, seizure, sweating, headache, vomiting, unknown, other.
- Onset of symptoms: < 1 h, >1 h, unknown (U).
- CPR surface: floor, spinal board, ambulance stretcher, bed, beach, U, others.

#### Variables collected by forensic pathologists

- Protocol for the SD study: protocol published previously [19].
- Worsheet for the study of resuscitation related injuries in forensic autopsies: published previously [20].

#### Variables collected by hospitals

. Admission date, location CA, hospital name 1, hospital name 2.

. Patient Data: Age, Sex.

- Coronary disease.
  Cardiac Catheterization: Y/N/Planned/U/Ongoing CPR, Catheterization: date/time, Significant coronary artery disease: left anterior descending artery (LAD)/right coronary artery (RCA)/circumflex artery (CA)/left coronary artery (LCA)/no, acute coronary thrombosis: LAD/RCA/CA/LCA/No.
- Treatment: Cardiac Surgery, Y/N/U; In Hospital Fibrinolysis Therapy, Y/N/U; Out of Hospital Fibrinolysis Therapy, Y/N/U; Prehospital Hypothermia, Y/N/U; Hospital Hypothermia, Y/N/U; Beta blockers, Y/N/U; Conterpulsation baloon pump, Y/N/U.
- Survival: admitted alive at the hospital, Y/N/U; discharged alive, Y/N/U; discharged destination, house/other sanitary center/others/U; 1 mouth Cerebral Performance Categories score (CPC), 1/2/3/4/5; 1 year CPC, 1/2/3/4/5/U; Death, Y/N/U.
- Clinical variables at the admittance: first average blood pressure, heart rate, Glasgow Coma Scale, temperature, glucose, oxygen saturation, fraction of oxygen inspired, gasometric analysis, lactate.
  Features of first ECG. Features of the initial echocardiogram.
- Others: Computed Tomography (CT) thoracic Y/N, CT cranial Y/N, mechanical compression received Y/N, thoracic lesions due to CPR Y/N, visceral lesions due to CPR Y/N.
- Causes of death: codified as ICD 9.

#### Variables from the medical background

- Patient data and epidemiological variables (nationality/residence/race/BMI).
- Cardiovascular risk factors.
- Cardiophaty background. (syncope, Sudden death family background, coronary disease, heart failure, valvulopathy, miocardiopathy, canalopathy, arrhythmia and other rhythm disturbances)
- ECG basal features.
- Vascular disease backgrounds.
- Broncophaty backgrounds. (Asthma, Chronic Pulmonary disease, Obstructive sleep apnea syndrome)
- No cardiovascular background (dementia scores/ osteoporosis and others).
- Medical treatment.

### Statistical analysis and sample estimate

The ReCaPTa statistical analysis of the data collected will be provided by a collaborator statistician. Prior to analysis all variables collected will be uniformly checked. A statistical software package will be used. The description of the variables studied will be done according to conventional techniques, with means and standard deviations for quantitative variables, and frequency tables for qualitative variables. Incidence rates of SCD will be calculated per 100,000 residents per month.

In the Camp de Tarragona, around 300 resuscitations were expected to be attempted by the EMS each year, including all ages and non-resident population. According to our previous results, autopsies were performed on 30 % of these patients. In 25 % of cases the patients were admitted to hospital with ROSC. To correctly estimate SCD rates we also include patients who presented a SCD and were not assisted by the EMS.

## Discussion

The methodology used to compile OHCA registries changes from country to country. In Spain, the Andalusian register uses an automatic and continuous registry based on the calls received and attendance provided by the EMS [25]. As in other registries such as the Swedish Cardiac Arrest Registry (SCAR) or the Osaka Utstein Project [26], all data is registered online by the EMS crew. The Cardiac Arrest Registry to Enhance Survival (CARES) collects and links data from 3 sources: 911 dispatch centers, EMS and receiving hospitals. This registry covers the whole United States and only enters events presumptively due to cardiac disease. It captures and compiles data from each source using multiple methods of data submission due to the diversity of participants [27].

In a systematic review of cardiac arrests attended by EMS in Europe, resuscitation attempts ranged between 38 and 86 per 100.000 inhabitants annually. This wide variation could be attributed to many factors such as the cardiac arrest risk, EMS activation, the threshold for initiating care and the case ascertainment [1].

There is an increase in the proportion of patients receiving successful defibrillation from bystanders with community AED before the arrival of the EMS [28]. A recent retrospective analysis of prospective cohorts included 13 OHCA registries non-randomly selected from different countries. It was found that 41.7 % of the registries were composed of enrolled patients exclusively treated by EMS personnel [29]. Our registry includes bystander CPR and bystander use of AED. Furthermore, 15.4 % of the cohorts included in this retrospective study were not periodically audited for missing cases and 23 % did not remediate the situation if missing cases were identified. The present study suggests that the EMS registries may present methodological variation due to different Utstein interpretations, case implementations and codifications. These produce different missing data magnitudes and it also suggests that the continuous monitoring of missing cases would increase the number of cases reported.

Strömsoe et al. found that out of 3198 OHCA cases in three counties in Sweden, 800 (25 %) were not reported prospectively by the EMS crew but were retrospectively discovered to be missing cases after a review of medical reports [30]. No single best way of assessing missing data has been put forward, which therefore makes it a potential new field for future research.

The ReCaPTa EMS database uses different sources of information to ensure quality control. These include codification numbers and data from the dispatch center, data from ALS medical records and data from BLS reports, thus providing valuable information for designing and building new quality control strategies.

The epidemiological study of SD requires the inclusion of all the OHCA assessed and non-assessed by the EMS. Consequently, the EMS database should be matched with the forensic database in order to minimize number of SCD cases that are overlooked.

SCD generally occurs in public places or at home which makes it difficult to evaluate initial information about symptoms and onset.

The Maastricht Study included SCD patients who had presented symptoms within 24 h prior to collapse [31]. Burke et al. performed autopsies on hearts with coronary disease from patients who had died suddenly within 6 h of the onset of symptoms [32]. As in other studies including multiple sources of information, ReCaPTa regards cases in which patients die within one hour of the onset of symptoms to be SCD [33].

Usually, the symptoms are evaluated by reviewing the medical report [10] or by family telephone interview [34], which can sometimes lead to incomplete data collection. To cope with this difficulty, our EMS database is based on an online application that is easily accessed and used by the crew attending the cardiac arrest and which records the variables of the onset and the types of symptoms.

In an SCD study performed in Germany, physicians collected bystander interviews on the scene immediately after the declaration of death or return of circulation [35]. The study evaluated symptoms from the 24-h period before the CA. Strikingly; it found that 25 % of patients presented no symptoms prior to collapse. This therefore means that evaluating the symptoms 1 h prior to collapse could provide relevant information.

Data reporting in our study is voluntary and can lead to missing cases as we note above. For this reason, the accuracy of our EMS database depends on continuous monitoring and providing regular feedback to the EMS crews.

In recent years monitoring studies have provided data from multiple sources. The ORE-SUD Study in Portland (US) reported an annual SCD incidence rate of 53 per 100,000 residents. Another similar methodological study performed in the west of Ireland found a quite similar incidence rate (51.2 /100,000).

Only partial data is available on the epidemiology of SCD in Spain [36]. SD has been traditionally associated with acute coronary disease [37]. However, a recent Spanish study based on autopsies showed that 21 % of patients presented a SD that was not associated with a cardiac cause. Compared with studies from US or northern Europe, patients with SCD presented a minor incidence of coronary disease and acute coronary thrombosis and the highest rate of cardiac hypertrophy among sudden death victims in the Mediterranean area [33].

Improved primary prevention strategies and the treatment of acute myocardial infraction (AMI) have probably decreased sudden death mortality secondary to coronary disease. However, SCD rates do not seem to have decrease in the last few years [38]. This situation, which may seem contradictory, has been attributed to an older population and a growing number of patients presenting SD secondary to non- acute coronary disease [3]. The incidence of AMI is lower in Spain than in countries in northern Europe or the United States [39], which means that our registry will make an important contribution to research into new preventive strategies and treatments for this emerging group.

One limitation of our study is the small population studied compared with other OHCA registries, although it is quite similar and some cases even larger than other studies that have monitored multiple sources of surveillance [10, 30]. The geographical area in our study receives many visitors, especially in the summer, and this could also be affect accurate estimates of SCD rates. Our database records each patient’s nationality and normal place of residence. Our SCD rates may have an upward bias because they include cardiac arrests among non-residents, but they may also have a downward bias because they do not include local residents who have developed SCD elsewhere [40].

Securing the collaboration of hospitals has traditionally been a challenge for OHCA studies. Our study involves researchers from three hospitals in the study area who were able to attend patients that had suffered an OHCA.

There have been no prospective studies in the Mediterranean area that include multiple sources of information for the study of SCD. Consequently, we believe that ReCaPTa could provide valuable information to deal with one of the greatest challenges facing modern medicine [41].

## Abbreviations

AED: Automated external defibrillator
AMI: Acute myocardial infraction
BLS: Basic life support
BMI: Body mass index
CA: Cardiac arrest
CA: Circumflex artery
CPC: Cerebral performance categories
CPR: Cardiopulmonary resuscitation
CT: Computed tomography
ECG: Electrocardiogram
EMS: emergency medical services
ICD: International code disease
ICU: Intensive care unit
LAD: Left anterior descending artery
LCA: Left coronary artery
LMFSIC: The Legal Medicine and Forensic Science Institute of Catalonia
No: N
OHCA: Out of hospital cardiac arrest
PEA: Pulseless electrical activity
PLR: Passive leg raising
RCA: Right coronary artery
ReCaPTa: Clinical and Pathological Registry of Tarragona
ROSC: Return of spontaneous circulation
SCD: Sudden cardiac death
SD: Sudden death
U: Unknown
US: United States
VF: Ventricular fibrillation
VT: Ventricular tachycardia
Yes: Y
